# Fear, depression, and well-being during COVID-19 in German and South African students: A cross-cultural comparison

**DOI:** 10.3389/fpsyg.2022.920125

**Published:** 2022-11-03

**Authors:** Rainer M. Holm-Hadulla, Claude-Hélène Mayer, Hannes Wendler, Thomas L. Kremer, Yasuhiro Kotera, Sabine C. Herpertz

**Affiliations:** ^1^Centre for Psychosocial Medicine, Heidelberg University, Heidelberg, Germany; ^2^Department of Industrial Psychology and People Management, University of Johannesburg, Johannesburg, South Africa; ^3^Department of Philosophy, University of Cologne, Cologne, Germany; ^4^Central Institute of Mental Health, Heidelberg University, Mannheim, Germany; ^5^School of Health Sciences, University of Nottingham, Nottingham, United Kingdom

**Keywords:** COVID-19, fear, well-being, depression, South Africa, Germany, cross-culture

## Abstract

Various studies have shown a decrease in well-being and an increase in mental health problems during the COVID-19 pandemic; however, only a few studies have explored fear, depression, and well-being cross-culturally during this time. Accordingly, we present the results of a cross-cultural study that (1) compares these mental health scores for German and South African students, (2) compares the correlations among them, and (3) identifies COVID-19 fear, well-being, and depression predictors. German and South African societies differ from each other socio-culturally, politically, and economically. Their university systems also differ to a large extent. University students in both countries completed the Fear of COVID-19 Scale, the World Health Organization-Five Well-Being Index (WHO-5), and the Patient Health Questionnaire-9 (PHQ-9). Welch’s *t*-test, correlation, and multiple regression analyses were performed. (1) German students were found to have statistically lower levels of COVID-19 fear and depression, but lower levels of general well-being than South African students. (2) In both samples, fear of COVID-19 was negatively correlated with well-being and positively associated with female gender and depression. (3) Additionally, female gender, depression, and lower well-being were identified as predictors of COVID-19 fear in both samples. The findings indicate that the fear of COVID-19 is associated with and varies according to gender, depression, and well-being across cultures, and that the difference in the intensity of fear between German and South African students may be partly explained by cultural and contextual differences. These findings can create a deeper understanding of the pandemic’s impact on student communities and may be used by mental health practitioners and researchers to develop and apply culture-specific interventions.

## Introduction

The COVID-19 pandemic has led to new ways of living, working, and studying, in a variety of social distancing and remote workplace scenarios. In a study in China with 746,217 participants, Ma et al. (2020) found indications of pronounced mental health problems in 45% of the respondents. In a North American study with 45,000 student participants, 35% of undergraduate students showed indications for major depression and 39% indicated generalized anxiety disorder (Chirikov et al., 2020).Comparable results were found in an extensive study in Germany (Holm-Hadulla et al., 2021). In light of this, the question concerning the influence of the COVID-19 pandemic on mental health is not only of global interest, but also of global character. Therefore, engaging with it calls for large-scale, global approaches.

Along these lines, we conducted a cross-cultural comparison between Germany and South Africa regarding fear, depression and well-being during the COVID-19 pandemic. Cross-cultural studies are of particular interest for understanding the changes in mental health during the COVID-19 pandemic. In a cross-cultural study between North America and Europe, increases in “anxiety, depression, headache and parafunctional oral behaviors” (Wieckiewicz et al., 2021, p. 1) were observed. In a comparison between the Middle East (Israel), Europe (Poland) and North America (Canada), Polish participants exhibited the greatest fear of infection, while Canadian participants worried more about their finances and relationships, as well as their physical and mental health, while Israeli participants were the least worried, generally speaking (Emodi-Perlman et al., 2021). A comparison between several Latin American and Caribbean Countries found an association between self-perceived level of concern regarding COVID-19 and gender, body-mass index and acquaintance with someone, who was infected with COVID-19 (Flores-Quispe et al., 2022). Despite their importance, cross-cultural studies are still rather rare. Therefore, they ought to be complemented by considering studies conducted within different cultural contexts: In Bangladesh, 28.5% of college and university students exhibited stress in general, 69.3% experienced event-specific stress, 33.3% suffered from anxiety and 46.9% were depressed during COVID-19 restrictions (Khan et al., 2020). Furthermore, fear of infection as well as socioeconomical and physical hardship were found to be positively associated with stress, anxiety, depression, and post-traumatic symptoms. In Spain, COVID-19 fear has shown to be associated with gender and anxiety in college student (Muyor-Rodríguez et al., 2021). A study conducted with college students from the Philippines found an association between information seeking behavior (particularly *via* Facebook), the local presence of COVID-19 cases and high levels of COVID-19 fear (Superio et al., 2021).

Our study contributes a novel perspective, since no investigation into fear, depression and well-being in Germany and South Africa has been advanced yet. However, we are also interested in identifying predictors for COVID-19 fear. A recurring pattern is that women score higher on COVID-19 fear. For instance, women under the age of 28,5, especially if they were single, less educated and lived in Europe, when compared to the USA (Wieckiewicz et al., 2021). In a study in Ecuador, female undergraduate students were the most vulnerable group regarding COVID-19 related fear, anxiety, stress and depression (Rodríguez-Hidalgo et al., 2020): According to Rodríguez-Hidalgo and colleagues’ model, COVID-19 fear and stress predict anxiety, which predicts depression. There is also a direct connection between stress and depression, but only a mediated one between COVID-19 fear and depression *via* anxiety (Rodríguez-Hidalgo et al., 2020). COVID-19 fear, however, concerns not just depression, but to other mental health issues as well. For instance, the intensity of COVID-19 fear is positively correlated with the rate of possible obsessive–compulsive disorder (Ji et al., 2020) and continuing use of tabacco or alcohol (Nguyen et al., 2020). Besides gender, socioeconomical and political variables have been used to predict vulnerable groups. Gallup (2020) found that 42% of parents of students in educational contexts are extremely worried about the negative effects of COVID-19 on their children’s well-being. A decrease in well-being and an increase in mental health problems were registered in medical and psychotherapeutic practices, counseling centers, and clinics in Germany (Holm-Hadulla et al., 2021). Moreover, increasing global racism (Ku, 2020) and inequality in general (Hess, 2020) are additional sources of stress for many individuals. The incidence of violence at home (Graham-Harrison et al., 2020) along with the experience of negative emotions such as shame (Vanderheiden and Mayer, 2021) have also increased, substantially interfering with individual well-being worldwide (Graves and Karabayeva, 2020). The COVID-19 impact on mental health and well-being has been reported as being substantial in previous studies (Li et al., 2021; Appleby et al., 2022). In a survey of five universities in Vietnam, older age, higher education, male gender, socioeconomical security and health literacy were identified as protective factors against COVID-19 fear (Nguyen et al., 2020).

This study focuses on students in Germany, as a highly industrialized country, and in South Africa as a developing country. The countries differ considerably in terms of their political and educational systems, socio-cultural implications, and economic standards, in addition to having disparate social norms, cultures and standards of living (Francesco and Gold, 2005). According to Hofstede et al. (2010), German culture tends to value adaptation to changing environments while South African cultural preferences are anchored in tradition. Further, optimism and indulgence are important in South African contexts, while pessimism is common in Germany (Hofstede et al., 2010). Various sources point out that culture influences the psychological well-being of individuals during COVID-19 (e.g., Sun et al., 2020; Kotera et al., 2020b, 2021b; Yap et al., 2021). These complex societal and cultural differences can become sources of stress affecting well-being (Rantanen et al., 2008). A study in 51 countries confirmed that the individual’s recent experience of well-being during COVID-19 is strongly connected to emotional concepts such as love, calm, determination, and loneliness (Sun et al., 2020). Central to feeling well during COVID-19 is the experience of control, connection, and calmness across cultures (Sun et al., 2020).

## The German university students’ context

The German university context has become increasingly multicultural during the past decades owing to immigration. Recent research has shown that in German students, the level of self-efficacy is relatively high in comparison to students who immigrated to Germany. German university students’ worldviews are associated with conservative and traditional views (Barz and Lieberwein, 2018) and a relatively high individuality index for German students without immigrant background in their family (Minkov et al., 2017). For German students, family, the educational system and peers are extremely important in terms of their own achievement and academic self-efficacy (Schunk and Meence, 2006). Verbal and emotional support also play a major role in helping students to advance in their studies (Schunk and Mullen, 2012). Furthermore, the peer group and peer support play a significant role in helping students to function within the educational system and in their personality and identity development.

Cyrulnik (2020) points out that in Germany, the practice of quarantine has evoked strong psychological negative consequences, even more in individuals with a history of early life stress. Further, it was reported on the WHO-5 that after one and a half years of rigid social restrictions and lockdowns, 72% of students in Germany believed their well-being had been seriously impaired (Holm-Hadulla et al., 2021). This corresponds to the finding that 75.8% of the students at a German university (the majority of German universities are governmental), showed indications of at least one syndrome diagnosis according to the PHQ-9. A study by Voltmer et al. (2021) found that between 2019 and 2020 there were only moderate consequences for mental health and well-being in the total German student group. Another study, however, already reported substantial experience with mental health problems at a German university in 2017 and 2018 (Koschig et al., 2021). Werner et al. (2021) observed a small increase in depression scales in German students during COVID-19 and no significant anxiety changes between 2019 and 2020. Karing (2021) researched depression, anxiety and stress in German students and found that 35.9% of the students showed a moderate to severe level of depression, 27.7% reported moderate to severe symptoms of anxiety, and 25.1% perceived high stress. Being female and older were risk factors for increasing mental health issues (Karing, 2021).

Fear associated with COVID-19 has been identified as a key construct in the poor mental health of German students amid the pandemic. German students were fearful of infection and stigma affecting their future and exacerbating their poor mental health (Herchenröder et al., 2020; Pauli et al., 2020). Spatafora et al. (2022) emphasize that fear of COVID-19 in German university students needs to be considered seriously as it can have a significant impact on their well-being.

In comparison with previous German studies, it is obvious that mental health issues increased massively during the pandemic. A study by Holm-Hadulla et al. (2009) showed that 20–25% of university students suffer from severe mental symptoms. Comparable results were shown in studies of Berger et al. (2015) and Kress et al. (2015).

## The South African university students’ context

The South African higher educational context is characterized by extreme social inequalities, slow transformation processes and ongoing racism (Adonis and Silinda, 2021). Since the end of apartheid in 1994, the educational system has been increasingly challenged by a growing diversity and heterogeneity of the student population, new educational system applications, changed institutional ideologies and challenged educators (Meier and Hartell, 2009). A South African value study (Fatoki, 2014) presented findings that achievement, self-direction, benevolence, security, universalism and conformity to a certain degree are important values for South African students.

Recent studies highlight the impact of the blurred boundaries between digital and physical worlds caused by remote working, technologization, digitalization, rapid changes, and stress-related problems. These situations involve insecure future perspectives, inadequate working conditions, and poor treatment in organizations which creates increased fear and stress, especially for employees in rapidly changing workplaces (Mayer and Oosthuizen, 2021a). The stress levels in South Africa are generally high and often lead to ill health in the population (van der Colff et al., 2009; De Beer et al., 2016; Mayer and Oosthuizen, 2021b). In addition to major stress experienced by staff in the educational sector (Jacobs and de Wet, 2015; Vos and Kirstens, 2015), university students suffer from severe depressive and anxiety disorders in comparison to the general South African population (Herman et al., 2009). In particular, students from low-income families struggle in their studies, experiencing high levels of stress (Letseka and Maile, 2008). The South African Stress and Mental Health survey which took place in 2008 estimated the prevalence of major depressive disorder at 8.9% and that of anxiety-related disorders at 14.6% (Herman et al., 2009).

The South African public’s general level of mental health declined during COVID-19 (Hedding et al., 2020; Zacher and Rudolph, 2020). About half of the South African population in a higher economic bracket met the diagnostic threshold of anxiety and depressive disorders: 46% and 47%, respectively, (De Man et al., 2022). One source of their mental distress was fear of getting infected with COVID-19. Another COVID-19 study reported that adults with childhood trauma were twice as likely to experience depression amid the pandemic (Kim et al., 2020). Moreover, 65% of children and young people in South Africa are reported to have experienced mental health issues (UNICEF, 2021). For young people in particular, the uncertain circumstances caused by COVID-19 have denied them hope and dignity, leading to anxiety and fear for the future (Heywood, 2021). Visser and Law-van Wyk (2021) found that students in South African universities reported psychological problems with high levels of anxiety (46%) and depression (35%) in coping with COVID-19, fearing losing control, being isolated and living a life on hold. These mental health difficulties affect their well-being during their studies (Visser and Law-van Wyk, 2021).

### Aim of the study

In a globalized world, students’ psychological problems and mental disorders afford reliable comparative data as a basis for internationally useful concepts of counseling (Holm-Hadulla et al., 2011; Holm-Hadulla and Koutsoukou-Argyraki, 2015; Koutsoukou-Argyraki et al., 2016). The concepts of fear, depression, and well-being need to be evaluated to provide students with support and increase mental health and well-being during and following the pandemic. Culture- and context-specific behavior can be understood and programmes to increase mental health and well-being can be developed only on the basis of empirical findings. Although previous studies compare Germany and South Africa in occupational populations (Kotera et al., 2021a), student comparisons have not been conducted in depth in the context of fear, depression, and well-being during COVID-19.

This study aimed to understand the fear of COVID-19 and the well-being of students in Germany and South Africa. First, the researchers compared the levels of COVID-19 fear, depression, and well-being between the two student samples (Aim 1). Second, the correlation among these variables between the two were compared (Aim 2). Finally, the researchers identified significant predictors of COVID-19 fear, well-being and depression in each sample (Aim 3).

In the following, fear, depression, and well-being research in the German and the South African student and university context are presented. Then, the research methodology is outlined and findings are presented and afterward discussed.

## Materials and methods

This study employed a cross-sectional design (Schmidt and Kohlmann, 2008) recruiting two samples of German students and South African students. The World Health Organization-Five Well-Being Index (WHO-5) and the Patient Health Questionnaire (PHQ-9) were administered to students from Germany and South Africa. Furthermore, students provided narrative reports.

### Participants

Notably, the German sample is the same as the one reported by Holm-Hadulla et al. (2021). Using a convenience sampling method, study invitations were sent to all students of Heidelberg University (27,162 students, 54.8% female, 45.2% male) *via* email with a link to participate in the study, which asked students to complete an online survey. The survey was conducted during a period in which the aforementioned social restrictions were in place, being available online from May 26th, 2021 up until June 11th, 2021 *via* the “LimeSurvey” platform. The response rate was 8.8% and, thus, the German sample consisted of 2,398 students (65.8% females [*n* = 1,578], 32.5% males [780], and 1.7 unanswered [40]; 27.6% age under 21 [662], 39.2% age 21–23 [941], 16.3% age 24–25 [392], 6.7% age 26–27 [161], and 10.1% age over 27 [242]). From these, 2,395 students (65.8% female [1578], 32.5% male [780], 1.7% [40] unanswered) completed the questionnaire and, consequently, were included in the analysis. South African students were recruited through emails and WhatsApp messages through lectures and professors as well as through the university research office. The South African sample consisted of 229 students (73.4% females [168], 25.3% males [58], and 1.3% unanswered [3]; 43.7% Age 1 [100], 32.8% Age 2 [75], 10.5% Age 3 [24], 4.8% Age 4 [11], and 8.3% Age 5 [19]). The recruitment period was 3 months in duration. Inclusion criteria included: being a student at the university at the time of the study. At least questionnaires of 200 students were required to fulfill the criteria. The sizes of both samples satisfied the required sample size calculated by power analysis (84: two tails, pH1(*r*) = 0.30, *α* = 0.05, power = 0.80, pH0 = 0; Faul et al., 2009). In the German sample, 66.8% (*n* = 1,603) were undergraduate and 33.2% (*n* = 795) were postgraduate students, whereas 90.4% (*n* = 207) of the South African sample were undergraduate and 9.2% (*n* = 21) were postgraduate students. Both samples included more female students than in the general university student population for each country. Normally 49% of students in Germany were female (Statista, 2022), and 56% of South African students were female (Statistics South Africa, 2017).

### Instruments

The original English language scales were used for the South African students, and the German language scales were used for the German students.

Students’ fear of COVID-19 was assessed using the Fear of COVID-19 Scale (Ahorsu et al., 2020), comprising seven statements regarding worries and anxiety associated with COVID-19. For example, “It makes me uncomfortable to think about Corona” was responded to on a five-point Likert scale (1 = strongly disagree to 5 = strongly agree). The German version of this scale was created in another study. Both scales demonstrated high internal consistency (English *α* = 0.82 [Ahorsu et al., 2020], German *α* = 0.93 [Seitz et al., 2021]).

Depression was measured using the PHQ-9 (Kroenke et al., 2001) and the German version of the PHQ-9 (Reich et al., 2018), consisting of nine items regarding the diagnostic criteria for major depressive disorder. The scale made statements about the state of respondents’ mental health during the past 2 weeks, such as “I have little interest or pleasure in doing things” using a four-point Likert scale (0 = Not at all to 3 = Nearly every day). Both scales demonstrated high internal consistency (English *α* = 0.86 [Kroenke et al., 2001], German = 0.79–0.88 [Gräfe et al., 2004]).

The WHO-5 was used to evaluate levels of well-being. This five-item scale appraises students’ subjective well-being with statements such as “I have been feeling cheerful and in good spirits” on a six-point Likert scale (0 = At no time to 5 = All of the time). WHO-5 demonstrated high internal consistency (English *α* = 0.86 (Omani-Samani et al., 2019), German *α* = 0.88 [Brähler et al., 2007]).

### Ethical considerations

Ethical approval was granted, both by the Ethics Committee of the University Hospital and the Data Protection Officer of Heidelberg University in Heidelberg, Germany, for the German dataset and by the Ethics Committee of the University of Johannesburg in South Africa for the South African data set.

### Statistical analysis

First, the data were screened for outliers and distribution. Measurement invariance tests were conducted to ensure that the comparisons could be made. Then, scores for fear of COVID-19, depression and well-being in the two student groups were compared (Aim 1). Because the sample sizes were substantially different and the assumption of homogeneity of variances was violated (Levene’s test for equality of variances *p* < 0.05), Welch’s *t*-tests were used. Next, correlation analyses were performed in each sample, to understand similarities and differences in correlations among these variables between the two student groups (Aim 2). Finally, multiple regression analyses were conducted to explore significant predictors for COVID-19 fear, well-being and depression in each sample (Aim 3). IBM SPSS version 27.0 was used to conduct all analyses.

## Results

### Comparing the levels of fear, depression, and well-being (Aim 1)

A Welch *t*-test was run to determine if there were differences in fear of COVID-19, depression and well-being between German and South African students (Aim 1; Table 1).

All three variables were significantly different between German and South African students (*p* < 0.001). South African students had a higher level of COVID-19 fear than German students (mean difference 6.46; 95% CI, 5.58–7.35; *t*(252.50) = 14.41; *p* < 0.001). The effect size was *d* = 0.43, indicating a medium effect size (Cohen, 1988).

South African students demonstrated a higher level of well-being than their German counterparts (mean difference 5.57; 95% CI, 1.99–9.15; *t*(256.85) = 3.06 *p* = 0.002). The effect size was *d* = 0.13, indicating a small effect size (Cohen, 1988).

German students showed a lower level of depression than South African students (mean difference 1.76; 95% CI, 0.77 to 2.75; *t*(258.80) = 3.49; *p* = 0.002). The effect size was *d* = 0.36, indicating a small to medium effect size (Cohen, 1988).

### Comparing the relationships between fear, depression, and well-being (Aim 2)

Correlation analyses were conducted in each sample (Table 2). Point biserial correlations were used for gender (0 = female, 1 = male) and the level of studies (0 = undergraduate, 1 = postgraduate).

**Table 1:** Comparing the levels of the fear of COVID-19, depression and well-being between German and South African students.

	German			South African			*t*-Value	CI 95%	*Cohen’s d*
	N	M	SD	N	M	SD			
Fear of COVID-19 [range: 5–35]	2,395	14.50	5.01	228	20.96	6.60	14.41***	5.58–7.35	0.43
Well-being (WHO-5) [range: 0–100]	2,358	37.56	21.27	229	43.13	26.71	3.06**	1.99–9.15	0.13
Depression (PHQ-9) [range: 3–27]	2,139	11.61	6.08	226	13.37	7.33	3.49**	0.77–2.75	0.36

Abbreviations: N, sample size; M, mean; SD, standard deviation; CI, confidence interval.

**p < 0.01; ***p < 0.001.

In the German sample, COVID-19 fear was negatively associated with well-being and positively associated with depression and female gender. Age and level of studies were not associated with COVID-19 fear. In the South African sample, COVID-19 fear was negatively associated with well-being and positively associated with depression and female gender. Level of studies and age were not associated with COVID-19 fear.

### Predictors of COVID-19 fear (Aim 3)

Multiple regression analyses were conducted to identify significant predictors of COVID-19 fear, well-being and depression. First, gender was entered to statistically adjust for its predictive effects (Table 2). Second, specified predictors of interest were entered accordingly. Adjusted coefficients of determination (Adj. *R*^2^) were reported. Multicollinearity was not a concern (Variance inflation factor < 10).

**Table 2:** Comparing the relationships between the fear of COVID-19, depression and well-being for German and South African students.

		German students					
		Age	Gender [0 = F; 1 = M]	Level of Studies [0 = UG; 1 = PG]	Fear of COVID-19 [range: 5–35]	Well-being (WHO-5) [range: 0–100]	Depression (PHQ-9) [range: 3–27]
South African students	Age	–	0.03	0.85**	0.03	0.04*	−0.05*
	Gender [0 = F; 1 = M]	0.08	–	0.03	−0.25**	0.06**	−0.11**
	Level of Studies [0 = UG; 1 = PG]	0.25**	0.01	–	0.02	0.03	−0.01
	Fear of COVID-19 [range: 5–35]	0.03	−0.23**	0.05	–	−0.20**	0.25**
	Well-being (WHO-5) [range: 0–100]	0.11	0.24**	0.06	−0.25**	–	−0.77**
	Depression (PHQ-9) [range: 3–27]	−0.07	−0.21**	−0.04	0.17*	−0.77**	–

Abbreviations: F, female; M, male; UG, undergraduate; PG, postgraduate. The values below the diagonal report correlations for the South African sample, while those above the diagonal report correlations in German sample.

*p < 0.05; **p < 0.01.

In both cohorts, female gender and well-being were predictors of COVID-19 fear; however, depression was a predictor among Germans, but not among South Africans. Gender was the strongest predictor of fear of COVID-19 in both cohorts (Table 3).

**Table 3:** Multiple regression predicting the fear of COVID-19 in German and South African students.

	Dependent variable: fear of COVID-19							
	German students				South African students			
	*B*	SE_B_	*β*	Value of *p*	*B*	SE_B_	*β*	Value of *p*
Step 1								
Gender [0 = F, 1 = M]	−2.41	0.20	−0.24	<0.001	−3.29	0.93	−0.23	0.001
Adjusted *R*^2^	0.06				0.05			
Step 2								
Gender	−2.17	0.21	−0.22	<0.001	−2.55	0.95	−0.18	0.008
Well-being (WHO-5) [range: 0–100]	−0.02	0.01	−0.09	0.005	−0.05	0.03	−0.21	0.037
Depression (PHQ-9) [range: 3–27]	0.13	0.03	0.16	<0.001	−0.02	0.09	−0.02	0.820
Adjusted *R*^2^	0.11				0.08			

Abbreviations: B, unstandardized coefficient; SE, standard error; *β*, standardized coefficient; F, female; M, male.

In both samples, female gender, fear of COVID-19 and depression were predictors of well-being; however, depression was the strongest predictor in both cohorts (Table 4).

**Table 4:** Multiple regression predicting well-being in German and South African students.

	Dependent variable: well-being (WHO-5) [range: 0–100]							
	German students				South African students			
	*B*	SE_B_	*β*	Value of *p*	*B*	SE_B_	*β*	Value of *p*
Step 1								
Gender [0 = F, 1 = M]	−2.87	0.88	−0.07	0.001	14.98	3.74	0.26	<0.001
Adjusted *R*^2^	<0.01				0.06			
Step 2								
Gender	−1.68	0.63	−0.04	0.008	5.69	2.55	0.10	0.027
Fear of COVID-19 [range: 5–35]	−0.18	0.06	−0.04	0.005	−0.37	0.18	−0.09	0.037
Depression (PHQ-9) [range: 3–27]	−2.62	0.05	−0.75	<0.001	−2.60	0.16	−0.72	<0.001
Adjusted *R*^2^	0.57				0.59			

Abbreviations: *B*, unstandardized coefficient; SE, standard error; *β*, standardized coefficient; F, female; M, male.

Only well-being significantly predicts depression in both cohorts; however, female gender and fear of COVID-19 are predictors of depression among Germans, but among south Africans (Table 5).

**Table 5:** Multiple regression predicting depression in German and South African students.

	Dependent variable: depression (PHQ-9) [range: 3–27]							
	German students				South African students			
	*B*	SE_B_	*β*	Value of *p*	*B*	SE_B_	*β*	Value of *p*
Step 1								
Gender [0 = F, 1 = M]	−1.38	0.26	−0.11	<0.001	−3.39	1.04	−0.21	0.001
Adjusted *R*^2^	0.01				0.04			
Step 2								
Gender	−0.67	0.18	−0.05	<0.001	−0.11	0.74	−0.01	0.883
Fear of COVID-19 [range: 5–35]	0.09	0.02	−0.07	<0.001	−0.01	0.05	−0.01	0.820
Well-being (WHO-5) [range: 0–100]	−0.21	<0.01	0.74	<0.001	−0.21	0.01	−0.76	<0.001
Adjusted *R*^2^	0.58				0.57			

Abbreviations: *B*, unstandardized coefficient; SE, standard error; *β*, standardized coefficient; F, female; M, male.

## Discussion

The present study compared the levels of COVID-19 fear, well-being, and depression in German and South African university students using the Fear of COVID-19 Scale, the WHO-5, and the PHQ-9. The analysis revealed German students had statistically lower levels of COVID-19 fear and of depression and lower levels of general well-being than South African students. In both samples, COVID-19 fear was negatively correlated with well-being and positively associated with female gender and depression. In both samples, female gender, depression and lower well-being were identified as predictors of COVID-19 fear. The findings indicate that the fear of COVID-19 is associated with gender and varies according to depression and well-being across cultures and that the difference in the intensity of fear between German and South African students may be partly explained by cultural and contextual differences. In the following paragraphs, the findings are discussed in the light of the literature review.

*German students have statistically lower levels of COVID-19 fear and depression, but lower levels of general well-being than South African students.*

The fear levels in the South African sample might be higher than in the German sample because South African students usually have to cope with extremely high stress levels caused by inequality (Hess, 2020), racism (Ku, 2020), economic pressure and poverty (Van Breda, 2018). Further, many of the South African adults and students come from very vulnerable and often poverty-stricken backgrounds (Department of Statistics, 2021), in which mostly female-headed households feel the experience of poverty (Department of Statistics, 2021). Because the first 2 years of the pandemic required the use of new technologies and remote work facilities, it may be assumed that South African students felt fearful, especially during the first year of COVID-19, since they often had to leave campus residences to return to home environments which were ill-equipped to handle the advanced technologies required for remote work (Vanderheiden and Mayer, 2021).

Additionally, it can be assumed that culture and context affects the level of fear experienced by students. German culture values the ability to adapt to changing environments, while South African cultural preferences are often anchored in tradition (Hofstede et al., 2010). It can be assumed that German students may have been able to adapt more easily and less fearfully to the new situation – not only because of their cultural background, but also because their living conditions generally seem to be more stable than the living conditions of South African students (Francesco and Gold, 2005; Snelgar et al., 2017).

Recent research (De Man et al., 2022) has also shown that South Africans are extremely anxious about possibly becoming infected with COVID-19. This fear may be stronger for South African than German students because many South Africans are likely to be aware that the health care systems and their personal and family circumstances might not allow for a successful COVID-19 treatment.

Since 65% of children and young people in South Africa experience mental health issues (UNICEF, 2021), it is unsurprising that the study at hand shows high fear levels and decreased well-being levels in South Africa, as supported by Heywood (2021) findings. The present study also corroborates the findings of Visser and Law-van Wyk (2021), and Makhubela and Mashegoane’s (2021) study which shows poor coping skills in students and high mental ill-health scores.

Furthermore, data were collected when excess mortality rates in South Africa reached a peak of over 16,000 per week (Sguazzin, 2022). Although the total number of deaths from COVID-19 has been determined to be approximately 110,000 in February 2022, the unofficial numbers are thought to be much higher (Sguazzin, 2022), with an excess of at least 300,000 COVID-19-related deaths in addition to the official numbers (Cowan, 2022). Official numbers of infection rates in South Africa during the 2 years of COVID-19 were the highest in Gauteng (NICD, 2022), the province in which the participating university is located. Research further shows that the excess mortality rate in South Africa was extreme (160%) in comparison to Western countries such as the UK and the US at around 50% (Heywood, 2021). This was probably based on different factors such as the very low vaccination rates, the overwhelmed health care system, the low rate of access to health care in selected groups of the South African population, and the high incidence of other diseases such as HIV, cardio-diseases, obesity and diabetes (Heywood, 2021; Sguazzin, 2022). In this context, it is not surprising that South African students are more fearful of COVID-19 than German students. Although the official numbers of deaths in Germany are similar to the official South African numbers (in February 2022 about 122,000 deaths in total in Germany), the excess death rate is very low in Germany in comparison to an average of 2000 excess deaths per week in South Africa in 2021.

The very high death rates in South Africa have terrified the population. This is likely to be one important reason why South African levels of fear are higher than German fear levels. Death during COVID-19 has become a very prevalent experience and seems to be much closer to South Africans than to Germans. This is especially the case when considering that the German population is around 83.2 million (DStatis, 2022), while South Africa counts only 60.4 million inhabitants (World Population Review, 2022). The high death rate, especially in the Gauteng province, has most probably caused extreme anxiety, also in students, since death has affected all populations groups and reduced the life expectancy at birth in South Africa in 2021 by 3.1 years for males and 3.8 years for females (Stats SA, 2021).

Further, in South Africa, there is no comparable social security system and hardly any social support within the society. Therefore, during COVID-19, the economic and financial impact during the lockdown periods were devastating for the families (Adebiyi et al., 2021) which might have led to higher levels of fear.

Research has shown that Germans during COVID-19 have placed high levels of trust in the government and federal decisions. This suggests a lower level of fear, because Germans have felt protected through governmental and political decisions (Riedl, 2020). In South Africa, however, the population has demonstrated declining levels of trust in the government during COVID-19. Research shows that South Africans were more likely to trust international organizations and doctors than their own politicians in good governance and decision-making (Wasserman and Madrid-Morales, 2021). This loss of trust in South Africans might also have contributed to higher levels of fear, while German students believed that the German government was effectively managing the disease (Riedl, 2020), and consequently experienced lower levels of anxiety. German students also may have felt more connected through digital devices and remote work, as described in Sun et al. (2020).

It is possible that digital communication might have compensated for the less dense social structure in Germany. In the South African context during COVID-19, many students returned to their family homes (Adebiyi et al., 2021) where they experienced family support and strong family ties and connections. Family units are also normally larger in South African families than in German families: the South African average household size is 3.3 individuals per household (ArcGIS, 2021), while in Germany it is 2.2 individuals per household on average and 38% are single households (Growth from Knowledge (GfK), 2017).

A South African value study by Fatoki (2014) reported that South African students value achievement, self-direction, benevolence, security, universalism, and conformity to a certain degree. These values may have helped South African students to stay focused on achievements and their personal self-direction during COVID-19 – although their learning environments changed and their family homes often did not cater for ideal learning conditions (Adebiyi et al., 2021). Student benevolence shown in supporting others during the times of crisis may have assisted in elevating their well-being. This hypothesis is supported by recent research which has shown that benevolence is negatively related to stress, mental ill-health and emotional exhaustion (Andersson et al., 2021).

Further, from a cultural point of view, South Africans usually demonstrate high levels of optimism, whereas the German population is often pessimistic (Kalberg, 1987). Recent studies show that pessimism has increased, particularly in younger generations in Germany recently (Schultheis, 2018). South Africans who are affected by continuing survival challenges still display high levels of optimism, which might have helped them during COVID-19 to have higher well-being than German students. Also, during COVID-19, South Africans were found to demonstrate more optimism than the global average by 14% (Lindique, 2021). This general optimistic attitude in 18- to 40-year-old South Africans may have kept students from having as low well-being as German students. Furthermore, South African students saw cooperation and knowledge exchange as key to overcoming the global challenges during the pandemic (Lindique, 2021). This solution orientation may have influenced their improved general well-being in comparison to that of German students.

*In both samples, COVID-19 fear was negatively correlated with well-being and positively associated with female gender and depression.*

Previous studies have pointed out that many individuals who experience high levels of fear are then unable to think healthily and logically and experience a decrease in well-being (Özmen et al., 2021). Therefore, COVID-19 fear might have also been negatively correlated with general well-being, as shown in this study in both samples. The study further supports previous studies which show that COVID-19 has a strong detrimental effect on mental health and well-being (Li et al., 2021; Appleby et al., 2022). This is the case in both the German and the South African samples and seems to be related to a rapid change in lifestyle, restrictions, social distancing, studying remotely, and associated changes as experienced globally during COVID-19 (Gallup, 2021; Tortorella et al., 2021). In addition, the students may have been exposed to high levels of violence at home (Graham-Harrison et al., 2020; McKinsey and Company, 2020). Such experiences could have influenced both samples and caused a decrease in general well-being.

COVID-19 fear was positively associated with female gender. This is not surprising, since research has shown that women, in particular, were hit by the COVID-19 pandemic on a global level in terms of employment losses, financial losses, gender-based violence, increased gender inequality, and vulnerability. “Increased childcare burdens, attitudinal bias, a slower recovery, or reduced public and private spending on services such as education or childcare make women leave the labor market permanently” (McKinsey and Company, 2020). The UN Deputy Executive Director Anita Bhatia (Lim, 2021) has emphasized that much of what women have fought for “could be lost in one year of Covid-19” since women (as nurses and health care workers) are more exposed than men to COVID-19, with a higher impact of disrupted access to health care, increased domestic violence, a strong push of women into poverty and a decreased global GDP which mainly affects women.

*In both samples, female gender, lower well-being and depression were identified as predictors of COVID-19 fear.*

The findings indicate that fear of COVID-19 is associated with gender, depression and general well-being across cultures. This suggests that women are predictably more fearful of COVID-19 than men, possibly because of their more vulnerable status in both German and South African societies. Furthermore, individuals with depression and a low sense of general well-being are also more vulnerable; their elevated levels of depression and reduced levels of well-being therefore predict COVID-19 fear. German and South African students might not have as many coping resources or support from their cultural groups or societies as men, and are therefore predictably more fearful than men and then anyone with a higher general well-being.

## Conclusion and recommendations

This study shows that German and South African students are being affected by fear, depression, and a reduced sense of well-being during COVID-19. However, the study also demonstrates how differences in the students’ fear, depression, and well-being are anchored in their different cultural, social, economic, political, and social contexts. In both countries, however, female students and students experiencing depression and a decreased general well-being are more vulnerable to COVID-19 fear.

Based on the findings, it is recommended that future research focuses on measures that could be taken to support students who are dealing with COVID-19 fear, depression, and lowered general well-being levels most effectively and culturally adapted. Accordingly, research could further explore the specific cultural factors and contexts which foster resilience and coping to support students in dealing with the short-term, medium-term and long-term effects of COVID-19.

Finally, on a practical note, counseling and therapy concepts and program can be developed based on these findings which directly address the impact of COVID-19 on students. The universities in Germany and South Africa can use these data to inform academics as well as administrative and support staff about the main psychological and emotional challenges of students during COVID-19. This could lead to the development of strategic professional program and services to support students in overcoming fears and depression and boosting their general sense of well-being. Female and vulnerable students with previously decreased well-being need to be specifically addressed in such program to prevent them from experiencing elevated levels of fear and depression.

## Limitations

As in every study, ours comes with certain limitations. First, although Welch’s *t*-tests were used to adjust the size difference, more equal samples should be used. Second, because self-report measures were used, response biases may also have been present (Kotera et al., 2020a). Third, as it was a cross-sectional study, causality of these variables was not evaluated.

Furthermore, recruitment was done at one university in each country; therefore, institutional bias may have been present. Especially in light of our convenience sampling method, it is possible that students who were particularly affected by the pandemic-situation responded more frequently to our study. This is in line with our finding that female students are more vulnerable to COVID-19 fear, which can help to explain why our study’s sample contained more female than male students. Nonetheless, this is also the case for comparable studies, for instance, from the USA (Chirikov et al., 2020) or China (Ma et al., 2020).

## Data availability statement

The raw data supporting the conclusions of this article will be made available by the authors, without undue reservation.

## Ethical statement

The studies involving human participants were reviewed and approved by Ethics Committee of the Medical Faculty of Heidelberg University. The patients/participants provided their written informed consent to participate in this study. The University of Johannesburg provided ethical consent for the South African study and the South African participants provided written informed consent.

## Author contributions

RH-H conceptualized the study and initiated it at the University of Heidelberg in 2021 where the study was pioneered together with SH. RH-H and his team at the University of Heidelberg collected the data set in Germany. C-HM collected the data at the University of Johannesburg. C-HM wrote up the introduction, the theoretical background and the discussion section. YK wrote up the research methodology and findings section and conducted the statistical analysis. RH-H, YK and HW contributed to the introduction and theoretical background. HW and TK contributed to revising the manuscripts. HW oversaw the revision process and substantially revised the introduction. TK substantially revised the methods and results sections. All authors had full access to all of the data in the study and take responsibility for the integrity of the data and the accuracy of the data analysis. All authors contributed to the article and approved the submitted version.

## Conflict of interest

The authors declare that the research was conducted in the absence of any commercial or financial relationships that could be construed as a potential conflict of interest.

## Publisher’s note

All claims expressed in this article are solely those of the authors and do not necessarily represent those of their affiliated organizations, or those of the publisher, the editors and the reviewers. Any product that may be evaluated in this article, or claim that may be made by its manufacturer, is not guaranteed or endorsed by the publisher.
